# Changes in bone turnover markers 6–12 months after bariatric surgery

**DOI:** 10.1038/s41598-024-65952-y

**Published:** 2024-06-27

**Authors:** Per G. Farup

**Affiliations:** Department of Research, Innlandet Hospital Trust, PB 104, 2381 Brumunddal, Norway

**Keywords:** Apolipoprotein E genotype (APOE), Bariatric surgery, Bone metabolism, Bone turnover markers, Obesity, Osteoporosis, Genetics, Biomarkers, Endocrinology

## Abstract

A rise in bone turnover markers (BTM) after bariatric surgery predicts poor bone health years later. This study explored factors associated with BTM and changes in BTM after bariatric surgery. Inclusion criteria were subjects 18 to 65 years of age with morbid obesity undergoing bariatric surgery. All data were measured before and 6 and 12 months after surgery. The study included 104 subjects: women/men: 83/21; mean age 43.1 (SD 8.4) years; BMI: 38.8 kg/m^2^ (SD 3.8). Surgery with Roux-en-Y gastric bypass (RYGB) and sleeve gastrectomy (SG) was performed in 84 (81%) and 20 (19%) subjects, respectively. From before to 6–12 months after surgery, procollagen type 1 N-terminal propeptid (P1NP) increased by 45.6 µg/L (95% CI 41.5–50.0, *p* < 0.001), and alkaline phosphatase (ALP) by 10 U/L (95% CI 7–14, *p* < 0.001). The increases were significantly larger after RYGB than after SG. The APOE- Ɛ3 allele was associated with low levels of BTM and high levels of leptin. There was an unfavourable increase in BTM after bariatric surgery. SG compared to RYGB and the presence of the APOE-Ɛ3 allele were associated with less unfavourable effects. The study emphasises the importance of optimal prophylactic interventions after bariatric surgery to prevent osteoporosis.

## Introduction

Reduced bone mineral density (BMD), osteoporosis and fractures are long-term side effects of bariatric surgery. Bone loss occurs as early as 6 months after surgery, and the increased fracture risk starts gradually three years after surgery and is increased by 21–44%^1,2^. Surgery with Roux-en-Y Gastric Bypass (RYGB) has, in several studies, been associated with a greater increase in bone turnover markers (BTM) and risk of fractures, and reduction of BMD than Sleeve Gastrectomy (SG)^1^.

BTM are products of the bone remodelling process, either markers of bone formation such as procollagen type 1 N-terminal propeptide (P1NP) and bone-specific alkaline phosphatase (ALP), or markers of bone resorption, such as carboxy-terminal cross-linked telopeptide type 1 collagen (CTX-1)^3,4^. They respond rapidly to changes in bone turnover but have limited specificity^3^. The rapid response makes them suitable for monitoring response to and compliance with treatment^3^. The role of BTM, either alone or combined into a risk score, as a predictor of fractures has been shown in several studies, reviews, and meta-analyses but is still not established^4–6^. It is, however, reasonable to believe that reducing BTM lowers the risk of osteoporotic fractures after bariatric surgery.

Changes in nutritional factors, gut hormones, oestrogen, adipokines, neuroendocrine hormones, fat and muscle mass, and reduced mechanical load due to weight loss result in poor bone health after bariatric surgery^1,7^. Knowledge about the importance of each factor associated with BTM is a prerequisite for implementing targeted treatment and thereby reduce the loss of BMD and the risk of fractures. This specific knowledge is, in large, missing.

The study aimed primarily to explore associations between BTM and changes in BTM from before to 6–12 months after bariatric surgery on one side and nutritional factors, endocrine changes, APOE genotypes, and surgical methods. The secondary aim was to study changes from before to after surgery in multiple variables related to bone health.

## Participants and methods

### Design

Data from the prospective cohort study MO-BiPS (Morbid Obesity—Bio-Psycho-Social disorders) were used^8^. Blood samples from a biobank were analysed and used in this retrospective cohort study.

### Participants

Subjects 18 to 65 years of age with morbid obesity (i.e. BMI > 40 or > 35 kg/m2 with complications related to obesity) referred by general practitioners to the Obesity Unit, Innlandet Hospital Trust, Gjøvik, Norway, from December 2012 to September 2014 for evaluation of bariatric surgery were asked for inclusion. Exclusion criteria were alcohol and drug abuse, previous major gastrointestinal surgery, and somatic and psychiatric comorbidity unrelated to obesity and in need of treatment. After inclusion, 6 months of preparation for surgery followed. During this period, there were regular follow-ups by doctors, nurses, and dieticians with information about the consequences of bariatric surgery, the need for dietary changes, weight loss, and physical activity. A strict "crispbread diet" or a meal replacement powder diet containing less than 4200 kJ/day was implemented in the last three weeks before surgery. Bariatric surgery was performed as RYGB or SG, decided by the surgeon in agreement with the subject^9,10^. After surgery, the participants were prescribed a multivitamin- and mineral supplement, calcium carbonate with vitamin D (1000 mg/800 IU) and oral iron (100 mg Fe^2+^ for men and post-menopausal women and 200 mg Fe^2+^ for pre-menopausal women) daily, and vitamin B12-injections every 3 months. Blood samples from immediately before surgery and 6 and 12 months after surgery were used in this study. More information about the participants and the design is given in previous publications^8,11^.

### Variables

The following variables were used. Abbreviations and reference values in brackets. The participants were asked to meet at the hospital in a fasting state in the morning, but persons living far away from the hospital might have had a small meal before leaving home.

- Demographic and anthropometric data: Age (years). Biological sex (male/female). Height (m). Body weight (kg). Body mass index (BMI; kg/m^2^).
- BTM (serum): Serum procollagen type 1 N-terminal propeptide (P1NP): (11–94 µG/L). Total serum alkaline phosphatase (ALP): (35–105 U/L). P1NP and bone-specific ALP are markers of osteoblast activity. Changes in total ALP related to changes in the liver-specific markers alanine aminotransferase (ALAT) and gamma-glutamyltransferase (gamma GT) were judged as changes in bone-specific ALP.
- Biochemistry (serum): C-reactive protein (CRP): (< 4 mg/L). Ionised Calcium (iCa): (1.15–1.33 mmol/L). Phosphate (Ph): (0.7–1.7 mmol/L). Magnesium (Mg): (0,71–0,94 mmol/L). ALAT: (10–70 U/L). Gamma GT: (10–115 U/L).
- Hormones: Free thyroxin (FT4): (8.5–16.5 pmol/L). Thyroid stimulating hormone (TSH): (0.44–3.1 mU/L). Leptin: (BMI < 25: women/men: 80–2500/ < 950 pmol/L). Adiponectin: (women/men: 4–22/2–20 mg/L). Parathyroid hormone (PTH): (1.2–7.1 pmol/L).
- Vitamins: Pyridoxal-5’-phosphate (Vitamin B6): (15–160 nmol/L). 25-Hydroxyvitamin D (Vitamin D): (37–131 nmol/L).
- Apolipoprotein E (APOE) genotypes: (*Ɛ*2*Ɛ*2, *Ɛ*2*Ɛ*3, *Ɛ*3*Ɛ*3, *Ɛ*2*Ɛ*4, *Ɛ*3*Ɛ*4, and *Ɛ*4*Ɛ*4). Genotyping was performed with allele-specific real-time polymerase chain reactions. Analyses unveiled significant differences between carriers and noncarriers of the APOE-Ɛ3 allele, and this post hoc dichotomisation was used in the analyses.

### Statistics

Descriptive data are reported as the mean (SD) and number with proportion (%). A linear mixed regression model for repeated analyses based on 10,000 bootstrap samples was used and reported as estimated marginal means or estimated coefficients (B-values) with 95% confidence intervals (percentiles) and p values. The changes in the variables from 6 to 12 months after surgery were judged as insignificant. Therefore, and to increase the power of the study, in the subjects with data from both 6 and 12 months after surgery, these results were combined in the analyses and compared with the data before surgery. The analyses were performed with IBM SPSS Statistics for Windows, version 27.0 (IBM Corp., Armonk, NY, USA).

## Results

Out of 152 correctly included subjects, 115 completed the lifestyle intervention and underwent bariatric surgery. This study included 104 subjects (women/men: 83/21 with a mean age of 43.1 (SD: 8.4) years, BMI: 38.8 kg/m^2^ (SD 3.8), with an APOE genetic test and at least one P1NP analysis. P1NP analyses were available in 96, 93 and 85 subjects before, and 6 and 12 months after surgery, respectively. Sixty-one subjects had leptin and adiponectin analyses before and 12 months after surgery. At inclusion, 20 (19%) were daily smokers, and 18 (17%) had diabetes. Surgery with RYGB and SG was performed in 84 (81%) and 20 (19%) subjects, respectively. The distribution of the APOE genetic variants was as follows: Ɛ2Ɛ2: 0 (0%); Ɛ2Ɛ3: 10 (10%); Ɛ3Ɛ3: 54 (52%); Ɛ2Ɛ4: 3 (3%); Ɛ3Ɛ4: 35 (34%), and Ɛ4Ɛ4: 2 (2%).

The BTM P1NP and ALP increased significantly after surgery and significantly more after RYGB than after SG. Most variables changed significantly from before to after surgery: Ph, vitamin D, and adiponectin increased, and CRP, vitamin B6, ALAT, gamma GT, FT4 and leptin decreased. Only the changes in P1NP and ALP were dependent on the surgical method. Table 1 gives the characteristics of the participants at inclusion and 6–12 months after surgery, comparisons between before and after surgery, and comparisons between the changes from before to after surgery with RYGB and SG.

**Table 1:** Participant characteristics and changes related to the point of time and surgical method.

Characteristics	Before surgery	After surgery (6–12 months)	Change (After minus Before)	Change differences (RYGB minus SG)
BMI (kg/m^2^)	38.8 (38.0; 39.6)	29.2 (28.5; 29.9)	− 9.6 (− 10.2; − 9.0) ***p* < 0.001**	− 0.64 (− 1.96; 0.72) *p* = 0.344
CRP (mg/L)	4.4 (3.9; 5.0)	1.5 (1.0; 1.9)	− 3.0 (− 3.7; − 2.4) ***p* < 0.001**	− 0.0 (− 1.6; 1.5) *p* = 0.957
iCa (mmol/L)	1.24 (1.24; 1.25)	1.24 (1.23; 1.25)	− 0.005 (− 0.011; -0.002) *p* = 0.186	− 0.00 (− 0.02; 0.01) *p* = 0.779
Phosphate (mmol/L)	1.00 (0.97; 1.03)	1.13 (1.10; 1.15)	0.13 (0.09; 0.16) ***p* < 0.001**	-0.03 (-0.11; 0.05) *p* = 0.461
Mg (mmol/L)	0.87 (0.86; 0.89)	0.86 (0.85; 0.87)	− 0.011 (− 0.027; 0.003) *p* = 0.163	0.02 (− 0.02; 0.05) *p* = 0.406
PTH (pmol/L)	5.47 (5.10; 5.84)	5.55 (5.22; 5.88)	0.08 (− 0.32; 0.48) *p* = 0.710	0.55 (− 0.28; 1.29) *p* = 0.190
Vitamin D (nmol/L)	61.4 (56.8; 66.1)	72.3 (68.1; 76.6)	10.9 (6.6; 15.4) ***p* < 0.001**	4.54 (-6.62; 15.68) *p* = 0.433
Vitamin B6 (p-PLP) (nmol/L)	60.5 (53.9; 67.2)	44.0 (38.1; 49.9)	− 16.6 (− 23.9; − 9.0) ***p* < 0.001**	− 2.4 (− 22.6; 20.9) *p* = 0.836
P1NP (microg/L) *	42.6 (38.4; 46.7)	88.2 (84.5; 91.8)	45.6 (41.5; 50.0) ***p* < 0.001**	15.1 (5.4; 24.9) ***p* = 0.005**
ALP (U/L)	67 (63; 70)	77 (74; 81)	10 (7; 14) ***p* < 0.001**	16.4 (9.3; 22.2) ***p* < 0.001**
ALAT (U/L)	37 (33; 41)	23 (20; 26)	− 14 (− 19; − 9) ***p* < 0.001**	− 2.1 (− 19.8; 12.5) *p* = 0.823
Gamma GT (U/L)	33 (29; 36)	18 (14; 21)	− 15 (− 19; − 11) ***p* < 0.001**	− 8.4 (− 14.0; − 2.4) *p* = 0.021
FT4 (pmol/L)	16.7 (16.1; 17.3)	15.4 (14.9; 15.9)	− 1.3 (− 1.9; − 0.6) ***p* = 0.001**	0.06 (− 0.85; 1.13) *p* = 0.920
TSH (mU/L)	1.61 (1.39; 1.83)	1.64 (1.44; 1.83)	0.03 (− 0.23; 0.27) *p* = 0.851	0.24 (− 0.21; 0.74) *p* = 0.361
Leptin (pmol/L)	2218 (2050; 2385)	1117 (949; 1284)	− 1101 (− 1340; − 852) ***p* < 0.001**	− 480 (− 1116; 239) *p* = 0.251
Adiponectin (mg/L)	7.7 (6.3; 9.1)	13.2 (11.7; 14.6)	5.4 (4.1; 6.8) ***p* < 0.001**	1.16 (− 1.95; 4.38) *p* = 0.495

Participant characteristics before and 6–12 months after surgery with comparisons between before and after surgery and comparisons of the changes from before to after surgery between the surgical methods. The analyses were performed with a linear mixed regression model based on 10,000 bootstrap samples adjusted for age, sex, point of time, type of surgery and APOE-Ɛ3 and are given as estimated marginal means, evaluated at the means of the covariates, with 95% confidence intervals (percentiles) and p values. Statistically significant p values in bold text.

*The increases in P1NP were statistically significant in both the RYGB and SG group (data not shown).

Four variables showed significant differences between subjects with and without the APOE-Ɛ3 allele. The levels of P1NP, ALP, and PTH were lower, and leptin was higher in subjects with the Ɛ3 allele than in those without. Table 2 gives the results. Other significant differences between subjects with and without the e3 allele were not found, and no changes from before to after surgery were dependent on the Ɛ3 allele (data not shown).

**Table 2:** Variables with significant differences between subjects with and without the APOE-Ɛ3 allele.

Variables	Ɛ3 (95% CI)	Not-Ɛ3 (95% CI)	Difference APOE-Ɛ3 (Ɛ3 minus not-Ɛ3)
PTH (pmol/L)	5.5 (5.1; 5.8)	6.5 (5.0; 7.9)	− 1.03 (− 1.63; − 0.50) ***p* < 0.001**
P1NP (microg/L)	64.3 (60.8; 67.8)	86.6 (70.8; 102.4)	− 22.3 (− 34.1; − 11.5) ***p* < 0.001**
ALP (U/L)	71.7 (68.4; 74.9)	78.4 (63.6; 93.2)	− 6.7 (− 10.9; − 2.7) ***p* = 0.001**
Leptin (pmol/L)	1685 (1564; 1806)	1318 (763; 1872)	368 (− 9; 708) ***p* = 0.002**

The analyses were performed with a linear mixed regression model based on 10,000 bootstrap samples adjusted for age, sex, point of time, and type of surgery and are given as estimated marginal means, evaluated at the means of the covariates, with 95% confidence intervals (percentiles) and *p*-values. Statistically significant *p*-values in bold text.

There was a significant positive association between P1NP and ALP (Table 3). Associations between the BTM P1NP and ALP and variables known to be associated with BMD and osteoporosis are given in Table 3. P1NP and ALP were positively associated with PTH. None of the associations between P1NP and ALP on one side and one by one of leptin, adiponectin, iCa, Ph, Mg, vitamin D, vitamin B6, FT4, and TSH were statistically significant.

**Table 3:** Associations between P1NP and ALP on one side and variables associated with osteoporosis.

Dependent variables						
	ALP (U/L)	Leptin (pmol/L)	Adiponectin (mg/L)	PTH (pmol/L)	iCa (mmol/L)	Phosphate (mmol/L)
P1NP (microg/L)	0.43 (0.29; 0.64) ***p* < 0.001**	0.002 (− 0.006; 0.006) *p* = 0.544	− 0.28 (− 0.86; 0.98) *p* = 0.492	2.58 (0.83; 4.08) ***p* = 0.001**	− 32 (− 150; 24) *p* = 0.528	2.7 (− 18.5; 20.7) *p* = 0.781
ALP (U/L)		− 0.002 (− 0.011; 0.003) *p* = 0.632	− 0.62 (− 1.49; 0.45) *p* = 0.189	2.12 (0.42; 3.80) ***p* = 0.017**	− 23 (− 107; 73) *p* = 0.610	− 4.8 (− 17.7; 8.3) *p* = 0.488

		Mg (mmol/L)	Vitamin D (nmol/L)	Vitamin B6 (nmol/L)	FT4 (pmol/L)	TSH (mU/L)
P1NP (microg/L)		8.7 (− 38.9; 50.8) *p* = 0.705	− 0.08 (− 0.24; 0.04) *p* = 0.259	0.00 (− 0.10; 0.10) *p* = 0.933	− 0.32 (− 1.86; 0.62) *p* = 0.527	− 1.39 (− 4.24; 0.84) *p* = 0.258
ALP (U/L)		30.9 (− 4.5; 65.6) *p* = 0.095	− 0.01 (− 0.11; 0.10) *p* = 0.872	0.016 (− 0.050; 0.097) *p* = 0.669	− 0.06 (− 1.12; 0.59) *p* = 0.883	− 1.28 (− 4.19; 0.65) *p* = 0.291

The analyses were performed with a linear mixed regression model based on 10,000 bootstrap samples with P1NP and ALP, one at a time, as dependent variables, and one by one of Leptin, Adiponectin, PTH, iCa, Ph, Mg Vitamin D, Vitamin B6, FT4 and TSH as independent variables adjusted for age, sex, point of time, surgical method, APOE-Ɛ3, BMI, smoking habits, diabetes, and CRP. Results are given as estimates (B-values) with 95% confidence intervals (percentiles) and *p*-values. Statistically significant *p*-values in bold text.

Associations between P1NP and ALP on one side and BMI, smoking habits, diabetes and CRP in addition to age, sex, point of time, surgical method, and APOE-Ɛ3 are given in Table 4. Age was positively associated with P1NP and ALP, and female sex was positively associated with ALP.

**Table 4:** Predictors of the bone turnover markers P1NP and ALP.

Dependent variable	Age (years)	Sex (male)	BMI (kg/m^2^)	Smoking (yes)	Diabetes (yes)	CRP (mg/L)
P1NP (microg/L)	0.34 (0.11; 0.65) ***p* = 0.005**	2.16 (− 4.62; 7.78) *p* = 0.414	− 0.29 (− 0.86;0.86) *p* = 0.552	− 0.65 (− 5.77; 4.98) *p* = 0.781	6.85 (0.51; 3.47) *p* = 0.019	− 0.78(− 1.91; 0.15) *p* = 0.128
ALP (U/L)	0.47 (0.22; 0.70) ***p* < 0.001**	− 5.62 (− 10.93; − 1.71) ***p* = 0.010**	0.26(− 0.45; 1.00) *p* = 0.477	2.53 (− 2.64; 6.68) *p* = 0.247	2.42 (− 1.79; 7.41) *p* = 0.244	0.37(− 0.82; 1.12) *p* = 0.469

Analysed with a linear mixed regression model based on 10,000 bootstrap samples with P1NP and ALP, one at a time, as dependent variables, and all at a time of age, sex, BMI, smoking habits, diabetes, CRP, point of time, surgical method, and APOE-e3 as independent variables. The results are given as estimates (B-values) with 95% confidence intervals (percentiles) and *p*-values. Statistically significant *p*-values in bold text.

## Discussion

The main findings were the significant increase in the P1NP and ALP 6–12 months after bariatric surgery. The strong association between P1NP and ALP indicates a common cause of the changes. The increases were significantly higher after RYGB than after SG, a difference that has also been reported in other studies^1,12–15^. Changes in other variables were not dependent on the surgical method. An interesting finding was the low levels of P1NP and ALP in subjects with the APOE-Ɛ3 allele. For the maintenance of good bone health after bariatric surgery, SG is preferable to RYGB, especially in subjects without the APOE-Ɛ3 allele.

P1NP and CTX-1 are elevated in subjects with osteoporosis and are recommended as biomarkers of bone formation and resorption, respectively^3^. Several studies indicate that P1NP and other BTM predict bone loss and progression toward osteoporosis and fractures^4–6^. Increased fracture risk and clinically overt osteoporosis take years to develop^1^. However, the detrimental effects of bariatric surgery on bone mass and microarchitecture are detectable 6 months postoperatively, which is in accordance with the rapid increase in BTM seen in this and other studies^2^. The differences in BTM after RYGB and SG agree with studies showing a reduction in BMD and increased risk of fractures after RYGB compared with SG^1,14–16^. SG is a purely restrictive procedure, while RYGB is a combined restrictive and malabsorptive procedure with a higher impact on fat and micronutrient absorption and bone health.

The associations between APOE-Ɛ3 and low values of BTM and PTH and high leptin values indicate a protective effect of the Ɛ3 allele on bone health. The role of APOE as a genetic risk factor for bone turnover, BMD, osteoporosis, and fractures in humans is inconclusive, which contrasts with the established role in mice^17,18^. The comprehensive meta-analysis by Lumsden et al. reports no significant associations between APOE genotypes and osteoporosis, osteopenia, pathological fractures or other disorders of the bone and cartilage^19^. In contrast, several studies have reported significant associations between APOE genetic polymorphisms and bone disease. Diekmann et al. reported increased levels of bone formation and resorption markers and reduced trabecular bone in Ɛ2 carriers, both in mice and humans, and concluded that the Ɛ2 allele is a genetic risk factor for low bone mass and fractures^20^. Other studies have reported associations between the Ɛ4 allele and hip fractures in a community-based study of older adults, reduced BMD in postmenopausal women with rheumatoid arthritis and the Ɛ4 allele, and reduced BMD and increased incidence of fractures in postmenopausal women with Ɛ2 and Ɛ4 compared with Ɛ3 carriers^21–23^. These findings agree with this study's favourable effects of the Ɛ3 allele. All Ɛ3-associated effects, low P1NP, ALP, and PTH and high leptin, are favourable for bone health.

Not only did P1NP and ALP change significantly after surgery. Changes in macro- and micronutrients, inflammatory markers, adipokines and gastrointestinal hormones are common after bariatric surgery and are involved in bone health^1,7,24,25^. In this study, Ph, vitamin D, and adiponectin increased, and CRP, ALAT, gamma GT, vitamin B6, FT4, and leptin decreased.

BTM were positively associated with age, female sex, PTH, RYGB, and absence of the APOE-Ɛ3 allele^26^. The associations with age and female sex were as expected. The prevalence of osteoporosis is higher in females than in men and increases with age. Secondary hyperparathyroidism, sometimes defined as an elevated PTH without elevated iCa, is a known complication after bariatric surgery^14,27^. This study gives no indication of secondary hyperparathyroidism. The expected decrease in iCa and increase in PTH were not observed, probably due to supplementation of vitamin D and Ca^7,27^.

The decrease in CRP and leptin and the increase in adiponectin after bariatric surgery and weight loss are well-known changes^25^. Leptin acts on bone metabolism through a central pathway, which inhibits bone formation and promotes resorption, and a peripheral pathway with opposite effects; the net result in most studies is a positive association between leptin and BMD^1,24,28^. Adiponectin and inflammatory markers such as CRP, which both putatively aggravate bone loss, changed in opposite directions after surgery^1,24^. The reduced levels of FT4, vitamin B6, and Mg, although not associated with BTM in this study, also affect bone health. Hypothyroidism increases bone mass and mineralisation, changes the bone structure, and increases the incidence of fractures^26,29^. Vitamin B6 is positively associated with BMD^30^. Smoking and low Mg values are related to osteoporosis, and Mg supplementation favours BMD and reduces fracture risk^31,32^. The lack of associations between BMI, smoking, diabetes, CRP, Mg, vitamin B6, FT4, leptin, adiponectin, CRP on one side and P1NP and ALP on the other could indicate that these factors affect bone health by mechanisms other than activation of BTM.

Impaired bone health after bariatric surgery occurs despite individual follow-up, nutritional advice and adequate supplementation of Ca and vitamin D. Pre- and postsurgery deficiencies in macro- and micronutrients (e.g. vitamins A, B1, D, K, and folate, iron, zink and selenium) are most likely involved in the pathogenesis^33,34^.

### Strengths and limitations

This study population, which was unselected subjects from the general population referred to the only obesity unit in the region, is representative of subjects undergoing bariatric surgery. The follow-up rate was satisfactory, and appropriate variables, including genetic markers, were used in the analyses. Most studies include both markers of bone formation and bone resorption. In this study, only markers of bone formation (P1NP and ALP) were used. CTX-1, a marker of bone resorption often measured in combination with P1NP, is sensitive to correct sampling and storing and was omitted because analyses in blood samples stored for ten years in a biobank might be unreliable. P1NP seems more sensitive to changes in bone metabolism than CTX-1^15,26^. Minor differences in the ratio between these two markers of bone formation and resorption could mean net bone gain or loss. Total ALP was used instead of bone-specific ALP. The increase in ALP and fall in ALAT and gamma GT after surgery render it likely that the increase in ALP is an increase in bone-specific ALP. The results based on the genetic marker APOE are encumbered with uncertainty because the dichotomisation into the APOE-Ɛ3 and non Ɛ3 groups was performed post hoc. The dichotomisation was one of several ways to dichotomise the variable, and the non Ɛ3 group was small. The results, which could be a type I error, were, however, in accordance with other reports^20–23^. The fact that most changes in the variables were within the normal ranges (the reference ranges) could question the clinical relevance of the changes. The identical pattern in the changes, which were highly significant, substantiates the clinical relevance. The use of a linear mixed regression model with bootstrapping is a recommended method for analyses of repeated measurements and reduces uncertainty about normality of the data.

## Conclusions

BTM increased significantly after bariatric surgery. The significant increase in P1NP and ALP 6–12 months after surgery, with a significantly higher increase following RYGB than SG, predicts bone loss with progression toward osteoporosis and fractures. High BTM (P1NP and ALP) was positively associated with female sex, age, and PTH, which are risk factors for osteoporosis and fractures. The presence of the APOE-Ɛ3 allele was associated with low BTM and PTH, and high leptin, which are favourable for bone health. The increase in adiponectin, and decrease in vitamin B6, FT4, and leptin were markers of unfavourable changes, whereas the increase in vitamin D and lower CRP were favourable changes. The changes in these variables, which affect bone health, were not associated with the BTM in this study and might act on other mechanisms. This study indicates that SG is preferable to RYGB for preserving good bone health after bariatric surgery. However, RYGB might have other metabolic advantages to SG that must be considered when choosing the type of surgery. The study emphasises the importance of optimal preventive intervention, especially in predisposed subjects, to avoid a detrimental effect of bariatric surgery on bone health. Supplementation with vitamin D, vitamin B6, and Ca is mandatory to avoid secondary hyperparathyroidism and micronutrient deficiencies.

### Ethics approval and consent to participate

The study was approved by the Norwegian Regional Committees for Medical and Health Research Ethics, PB 1130, Blindern, 0318 Oslo, Norway (reference number 2012/966 with an amendment of June 28, 2018). The study was performed in accordance with the Declaration of Helsinki. Written informed consent was obtained from all participants before inclusion.

## Data Availability

The raw datasets generated and analysed during the current study are not publicly available to protect participant confidentiality. Case report forms (CRFs) on paper are safely stored. The data were transferred to SPSS for statistical analyses, and the data files are stored by Innlandet Hospital Trust, Brumunddal, Norway, on a server dedicated to research. The security follows the rules given by The Norwegian Data Protection Authority, P.O. Box 8177 Dep. NO-0034 Oslo, Norway. The data are available on request to the author.

## Abbreviations

ALAT: Alanine aminotransferase
ALP: Alkaline phosphatase
APOE: Apolipoprotein E genotypes
BMD: Bone mineral density
BMI: Body mass index
BTM: Bone turnover markers
CTX-1: Carboxy-terminal cross-linked telopeptide type 1 collagen
CI: Confidence interval
CRP: C-reactive protein
Gamma GT: Gamma-Glutamyltransferase
FT4: Free thyroxin
iCA: Ionised Calcium
Mg: Magnesium
P1NP: Procollagen type 1 N-terminal propeptide
Ph: Phosphate
PTH: Parathyroid hormone
RYGB: Roux-en-Y Gastric Bypass
SD: Standard deviation
SG: Sleeve gastrectomy
TSH: Thyroid stimulating hormone
Vitamin B6: Pyridoxal-5’-phosphate
Vitamin D: 25-Hydroxyvitamin D
